# The Good Old Masserann Technique for the Retrieval of a Separated Instrument: An Endodontic Challenge

**DOI:** 10.7759/cureus.45811

**Published:** 2023-09-23

**Authors:** Utkarsh Umre, Shweta Sedani, Pradnya P Nikhade, Abeer Mishra, Akansha Bansod

**Affiliations:** 1 Conservative Dentistry and Endodontics, Sharad Pawar Dental College and Hospital, Datta Meghe Institute of Higher Education and Research, Wardha, IND; 2 Prosthodontics, Sharad Pawar Dental College and Hospital, Datta Meghe Institute of Higher Education and Research, Wardha, IND

**Keywords:** endodontic mishap, ortho-grade removal, masserann kit, instrument retrieval, separated instrument

## Abstract

Instrument separation is one of the most upsetting endodontic errors, and it can occur at any time during root canal surgery. A broken file causes a metallic restriction in the root canal and makes it difficult to clean and shape the instrument effectively, thereby making the prognosis questionable. Hence, such a fragment should be properly retrieved when it becomes difficult to bypass it. A variety of techniques and tools are utilized for instrument recovery; however, most of them are expensive, difficult to master, and technique-sensitive. One such tool for orthograde removal of intracanal metallic obstacles is the Masserann kit. We discuss a case involving file removal from the maxillary premolar by using the Masserann technique.

## Introduction

Instrument separation is very common in root canals, with fascinating anatomical variations that make the treatment more difficult. These anatomical differences in the canal put patients at risk of a range of iatrogenic issues, including missing canals, instrument separation, gouging, perforation, and excessive extension of the obturation materials. One such unfavorable occurrence is instrument breakage, which may impede the cleaning and shaping processes and cause pain or discomfort [1]. Instrument separation is one of the most common reasons for the failure of root canal treatments. The most frequent causes of file separation are incorrect use, insufficient access, root canal anatomy, and even manufacturing flaws [2]. Instrument separation is a highly distressing incident, and it has an incidence rate of 2-6% during endodontic therapies. On rare occasions, a detached instrument in a canal system may obstruct entry to the apex during nonsurgical root canal therapy [3]. Therefore, before obturating to the level of separation or beginning surgery, an attempt should be made to bypass or retrieve the tool. Orthograde retrieval is frequently challenging and time-consuming, with a success rate of 55-79% [4].

There are various methods and tools to retrieve separated instruments, such as manual instrumentation, ultrasonic technology, canal finder systems, Masserann kits, the Endo-Extractor System, and various pliers as well as chemical agents like iodine trichloride. In particular, the dental operating microscope validates the proverb "If you can see it, you can probably do it," by enabling physicians to see most broken devices [5]. The Masserann method is associated with an overall success rate of 55%, and it is beneficial for removing posts, silver points, and damaged files from the root canal [6]. In this report, we present a case where the Masserann technique was used to successfully remove a separated file that was lodged in the apical to the middle one-third region of the buccal canal of the maxillary left first premolar.

## Case presentation

**Figure 1:**
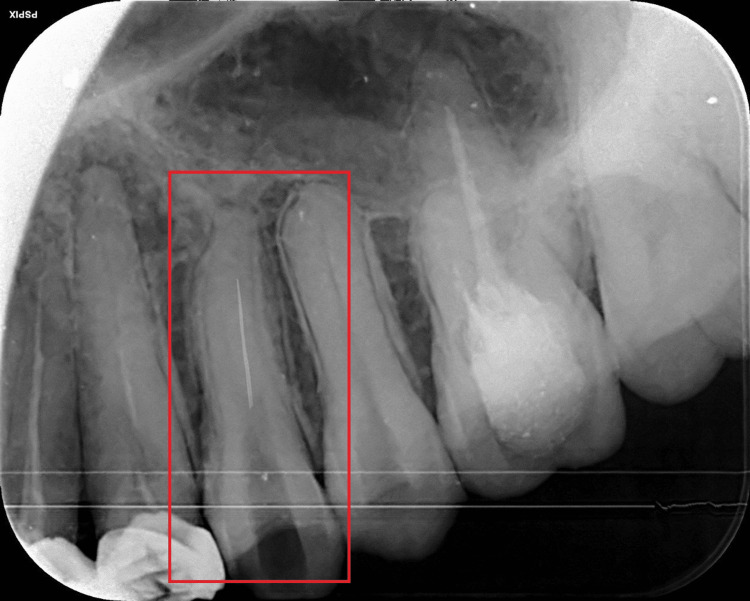
Radiographic confirmation of the separated instrument in 24 Preoperative radiograph of the separated instrument in 24

A 55-year-old female patient presented to the Department of Conservative Dentistry and Endodontics, Sharad Pawar Dental College and Hospital; she had been referred from the Prosthodontic Department for intentional root canal treatment of the upper right first premolar (tooth number 24) and second premolar (tooth number 25). Access opening was performed with a No. 1 endo access bur in 24 and 25 following the administration of local anesthesia. Both 24 and 25 contained two canals, the buccal and palatal canals. The working length was established and the cleaning and shaping of the root canal procedure were initiated. A radiograph was acquired to confirm the separation of the W3-path (12/.vt) (Endo Plus, Woodpecker), which was separated in the buccal canal in 24 during cleaning and shaping, as shown in Figure 1.

**Figure 2:**
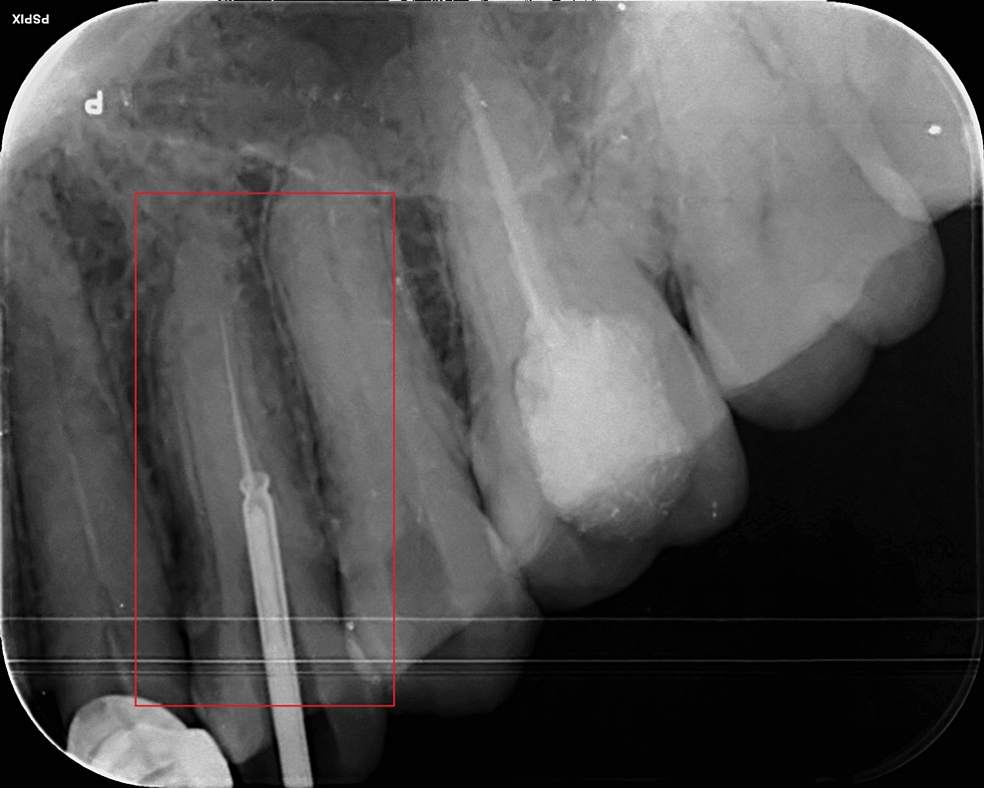
Radiographic confirmation of the location of the extractor in 24 The extractor was seen encircling 2 mm of the separated fragment in 24

At the intersection of the middle third and apical third of the canal, around 6 mm of the instrument got broken. Because bypassing the fragment was unsuccessful due to the separated file's length of more than 5 mm approximately, the Masserann technique was employed for instrument retrieval. Utilizing diamond points, the coronal access in tooth 24 was improved (Shofu Preparation Kit, Shofu, Kyoto, Japan). The consecutive application of the Peesso reamer straightened the obstruction's radial access (Mani Inc., Utsunomiya, Japan). End-cutting trephan with an acceptable diameter was chosen by superimposing it over the obstruction on the radiograph as the size of the obstruction was unknown. To remove the radicular dentin surrounding the broken instrument, the trephan was slowly spun in an anticlockwise direction. Radiographical monitoring was done to see how far the trephan had advanced. A 1.2-mm diameter extractor tube was inserted into the canal to encircle and hold the obstruction once the coronal section of the impediment was released from the dentin. After radiographic confirmation that the fragment was completely encircled by the extractor tube, the obstruction was tightly gripped against the extractor tube's wall by rotating the plunger rod of the tube in a clockwise manner, as shown in Figure 2.

**Figure 3:**
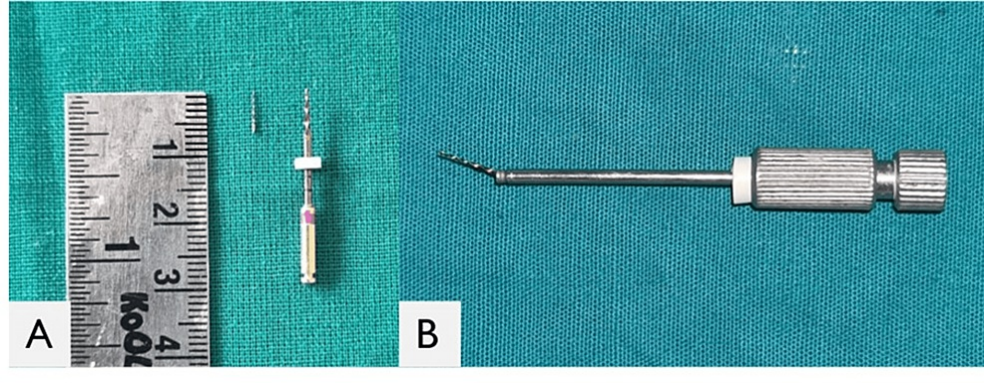
Image of the separated fragment after retrieval A: The retrieved fragment was 6 mm in length. B: The separated fragment was found lodged in the extractor of the Masserann kit

The extractor tube was gently twisted back and forth to loosen and remove the obstruction from the canal once tactile gripping of the impediment was confirmed. The separated fragment was successfully retrieved, as shown in Figure 3.

**Figure 4:**
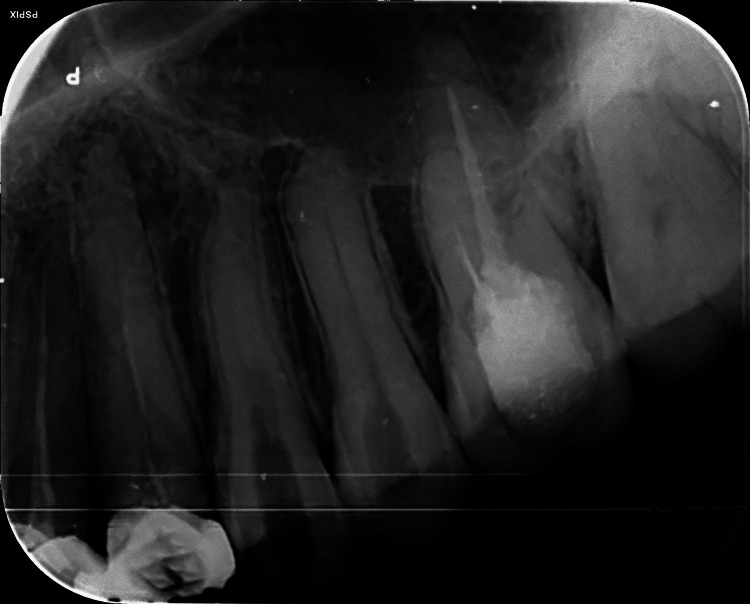
Postoperative radiograph after the removal of the separated fragment in 24 Confirmation of the retrieval of the separated fragment in 24 was done using a radiograph

Radiographic evidence supported the removal of the blockage from the canal of tooth 24, as shown in Figure 4.

**Figure 5:**
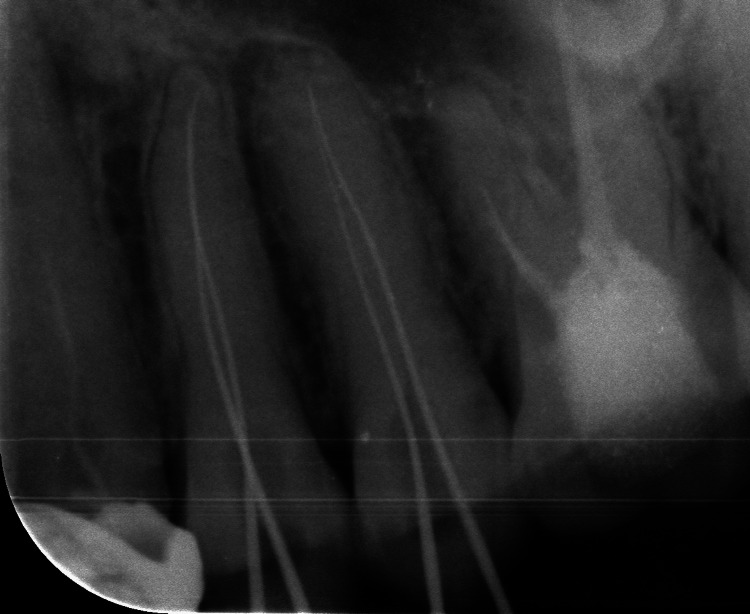
Working length measurement in 24 and 25 Radiograph showing working length measurement in 24 and 25 using a size 10 K file. The working length was found to be 21 mm

After the impediment was removed from tooth 24, a ledge was found apical to the area of the obstruction. A size 10 K file was utilized to navigate the cliff. The pulp space was further investigated, and another canal was found. After canal negotiations, the working length was calculated using radiography and the apex locator (J. Morita USA Inc., Irvine, CA) (Figure 5).

**Figure 6:**
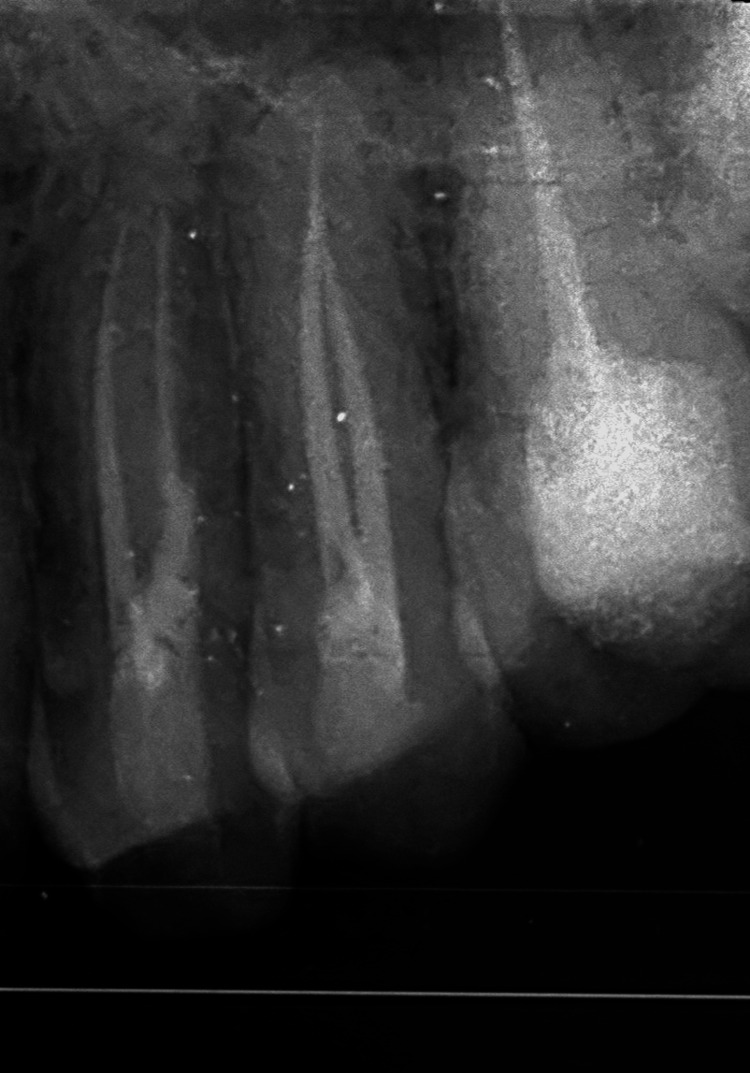
Postoperative radiograph of obturation and post-endodontic restoration of 24 and 25 Postoperative radiograph showing obturation and post-endodontic restoration of 24 and 25

NiTi files were used to form and clean canals with the crown-down technique (ProTaper, Dentsply Maillefer, Ballaigues, Switzerland); 2.5% sodium hypochlorite and 2% chlorohexidine were used for irrigation. As an intracanal medication, calcium hydroxide (Calcicur, VOCO GmbH, Cuxhaven, Germany) was employed. Using Gutta percha points (ProTaper, Dentsply Maillefer), Dia-Proseal sealer (Diadent, Burnaby, Canada), and single cone technique, the canals of tooth 24 were sealed on the subsequent visit. Composite was used to repair the access aperture, as shown in Figure 6, and post-endodontic therapy was advised for the patient.

## Discussion

Instrument breakage prevents proper chemomechanical preparation in root canals, which may negatively affect the treatment procedures. The fragment also inhibits the apical end of the root canal. Rotating instruments can break due to a number of reasons, including the operator's lack of experience, the device's speed and torque, curvature, instrument design and technique, and manufacturing flaws [7]. The management of separated instruments ultimately aims to protect the integrity of the dental structure. Separated instruments typically make it difficult to reach the root apex in a straight line, which makes it challenging to clean, disinfect/sterilize, and fill the whole root canal system [8].

The best course of action when an instrument breaks, according to Machtou and Reit, is to retrieve it [9]. However, no definitive procedure has been established to extract a broken instrument from the root canals, according to the literature. Although there are many specialized instrument retrieval kits and systems available, they come with their drawbacks, such as excessive dentin removal from the root canal, ledging, perforation, limited use in roots that are narrow and curved, and extrusion of the fractured portion through the apex [10]. As a result, the dentist must decide whether to try to remove the instrument, avoid it, or leave a separate part in the root canal. Rocke and Guldener recommended considering several variables before making a decision, including the pulp state, per-apical infection, the canal anatomy, the position of the broken file, and the fractured instrument [11].

Instrument recovery using the Masserann kit is successful in the anterior and posterior regions, with success rates of 73% and 44%, respectively [12]. This tool consists of 14 hollow cutting-end trephine burs (sizes 11-24) with a diameter of 1.1-2.4 mm and two extractors. The dentine around the detached instrument is removed by rotating the trephine counterclockwise. The primary benefit of the Masserann kit is the speedy removal of the detached instrument without the need for heating or pushing the fragment further apically. It enables the fractured instrument's outer edges to release [13]. Instrument retrieval is now generally achievable because of technological advancements and the availability of magnifying tools. The damage to the canal dentine is reduced by using a microscope or magnifying loupes to direct the retrieval of the instrument. According to Nevares et al., when the separated fragment was visible under a dental microscope, the retrieval success rate was 85.5%, as opposed to 47.7% when the fragment was not visible [14].

According to Ruddle, a staging platform can be created by choosing a Gates Glidden drill whose maximum cross-sectional diameter is marginally greater than the instrument being visualized. The Gates Glidden drill's bud is modified by cutting it perpendicular to its long axis at the diameter of its largest cross-section. This staging platform aided in the introduction of ultrasonic instruments for the safe removal of the peripheral dentin surrounding the exposed fragment, which improved the instrument's hold with the extractor and permitted the retrieval of the fragment [15].

In our case, the separated instrument was tightly lodged in the apical third of the maxillary first bicuspid. Since attempts to get around it were unsuccessful, the Masserann method was utilized. The trephan's centering over the piece was made easier by having direct access to it. By carefully cutting the peripheral dentin surrounding the fragment, this assured circumferential release of the coronal end of the fragment. This provided a firm grasp on the fragment and helped retrieve it along the long axis of the root, enabling routine retreatment.

## Conclusions

To minimize instrument separation and the accompanying stress and anxiety, proactive prevention should be the primary focus. In cases of separation, opting for safe retrieval or bypassing techniques is essential. The Masserann method is a retrieval procedure that demands both time and effective risk management. Its application is best suited for the extraction of firmly lodged metallic obstructions found in premolars and other posterior teeth and identified as separated instruments. However, it should be limited to straight canal segments with ample surrounding dentin, as it is only effective in such areas and necessitates a high level of clinical skill and unobstructed access along a linear path.
